# NONHUMAN PRIMATE GUT MICROBIOME: IMPACT OF AGE AND CALORIE RESTRICTION

**DOI:** 10.1093/geroni/igz038.3073

**Published:** 2019-11-08

**Authors:** Ricki Colman, Federico Rey

**Affiliations:** University of Wisconsin, Madison, Wisconsin, United States

## Abstract

The human microbiome is composed of bacteria, archaea, viruses and eukaryotic microbes that reside in and on our bodies, the largest community of which is in the gut. Although the functions of the gut microbiota are not fully understood, they are known to play an essential role in immune, endocrine, and metabolic functions. To begin to understand the relationship between the gut microbiome, aging, and adult-onset, moderate calorie restriction in rhesus macaques (Macaca mulatta), we collected fecal samples at one timepoint from a total of 52 macaques for 16S rRNA gene analyses. Samples were taken from 20 males and 20 females across the natural macaque age range and from 6 males and 6 females enrolled in the long-term study of aging and calorie restriction at the Wisconsin National Primate Research Center. Preliminary data show that, like humans, NHPs exhibit a large interindividual variation in microbiota composition despite well-controlled environmental conditions.

